# Selection and Mechanism Study of Q-Markers for Xanthocerais lignum Anti-Rheumatoid Arthritis Based on Serum Spectrum–Effect Correlation Analysis

**DOI:** 10.3390/molecules29133191

**Published:** 2024-07-04

**Authors:** Hao Qian, Lei Su, Yaqiong Yang, Xiangyang Tian, Qingge Dai, Fantao Meng, Xiaoqin Wang

**Affiliations:** Department of Pharmacy, Inner Mongolia Medical University, Hohhot 010110, China; qianhao0@stu.immu.edu.cn (H.Q.); 15248255098@163.com (L.S.); yang0041@stu.immu.edu.cn (Y.Y.); tianxiangyang@stu.immu.edu.cn (X.T.); 15771378630@163.com (Q.D.); 13141797213@163.com (F.M.)

**Keywords:** Xanthocerais lignum, rheumatoid arthritis, UPLC-Q-Exactive Orbitrap MS, serum spectrum–effect correlation analysis, Q-Marker

## Abstract

Objective: To elucidate the chemical profile of Xanthocerais lignum’s extracts of different polarities and their impact on rheumatoid arthritis (RA), we identified anti-RA markers and predicted their action mechanisms. Methods: A collagen-induced arthritis rat model was established, and UPLC-Q-Exactive Orbitrap MS technology was employed to analyze and identify the chemical constituents within the alcohol extract of Xanthocerais lignum and its various extraction fractions, as well as their translocation into the bloodstream. Serum spectrum–effect correlation analysis was utilized to elucidate the pharmacodynamic material basis of Xanthocerais lignum against RA and to screen for Q-Markers. Finally, the potential anti-RA mechanisms of the Q-Markers were predicted through compound-target interaction data and validated using molecular docking techniques. Results: We identified 71 compounds, with flavan-3-ols and flavanones as key components. Of these, 36 were detected in the bloodstream, including 17 original and 19 metabolized forms. Proanthocyanidin A2, dihydroquercetin, catechin, and epicatechin (plus glucuronides) showed potential anti-RA activity. These compounds, acting as Q-Markers, may modulate ERK, NF-κB, HIF-1α, and VEGF in the HIF-1 pathway. Conclusions: This research clarifies Xanthocerais lignum’s pharmacodynamic material basis against RA, identifies 4 Q-Markers, and offers insights into their mechanisms, aiding quality assessment and lead compound development for RA treatment.

## 1. Introduction

Rheumatoid arthritis (RA) is an autoimmune disorder primarily characterized by erosive, symmetrical polyarthritis, frequently afflicting smaller joints such as those in the hands and feet, and potentially extending to extra-articular systems, even culminating in joint deformities and functional impairment [1]. Untreated, RA significantly diminishes patients’ quality of life and life expectancy. The global incidence of RA is approximately 0.5% to 1.0%, with a peak onset age between 30 and 50 years, and the prevalence in females is roughly 3 times that of males [2].

Currently, the scientific community is actively engaged in the quest for alternative natural remedies with anti-RA properties derived from plants, animals, and microorganisms. These medicinal sources exhibit a wide range of diversity and abundance, coupled with minimal adverse effects. Studies on their mechanisms of action have revealed that many of their constituents possess anti-inflammatory and immunomodulatory properties, offering distinctive advantages in the treatment of RA [3,4]. Xanthocerais lignum is the dried stem or branch wood of *Xanthoceras sorbifolia* Bunge in the Sapindaceae family, as recorded in classical Mongolian and Tibetan medical texts such as “*Ren Yao Bai Jing Jian*”, “*Wu Wu Meng Yao Jian*”, “*Jing Zhu Ben Cao*”, and “*Meng Yao Zhi*”. It is known for its efficacy in “*Zao Xie Ri Wu Su*”, clearing heat, reducing swelling, and alleviating pain, primarily used clinically to treat RA and rheumatic internal heat [5,6,7].

Quality markers (Q-Markers) in traditional Chinese medicine are indicative substances reflecting the safety and efficacy of Chinese herbs, based on the core theory of “Chinese medicine efficacy—pharmacodynamic material basis—quality control characteristic components” [8]. Chinese medicine, a complex multi-component system, often presents challenges in identifying and analyzing its pharmacodynamic material basis using traditional methods. Serum spectrum–effect correlation analysis is an emerging research approach that considers the metabolism and transformation of Chinese medicine within the body, identifying the translocated components of Chinese medicine in the blood and correlating them with pharmacological data to pinpoint chromatographic peaks associated with efficacy [9]. This method not only reveals the synergistic actions of multiple targets and components inherent in Chinese medicine but also embodies the holistic perspective of traditional Chinese medicine. The anti-RA therapeutic efficacy of Xanthocerais lignum is well-established, yet its pharmacodynamic material basis and mechanisms of action remain unclarified. Therefore, this study aims to elucidate the chemical constituents and constituents absorbed into the blood of Xanthocerais lignum using UPLC-Q-Exactive Orbitrap MS technology and to perform serum spectrum–effect correlation analysis with its anti-RA efficacy in order to screen for anti-RA Q-Markers of Xanthocerais lignum. Furthermore, this study predicts the mechanisms of action of these Q-Markers based on compound–target interaction data and validates them through molecular docking techniques.

## 2. Results

### 2.1. Chemical Constituents of Various Extracts of Xanthocerais lignum

**Figure 1:**
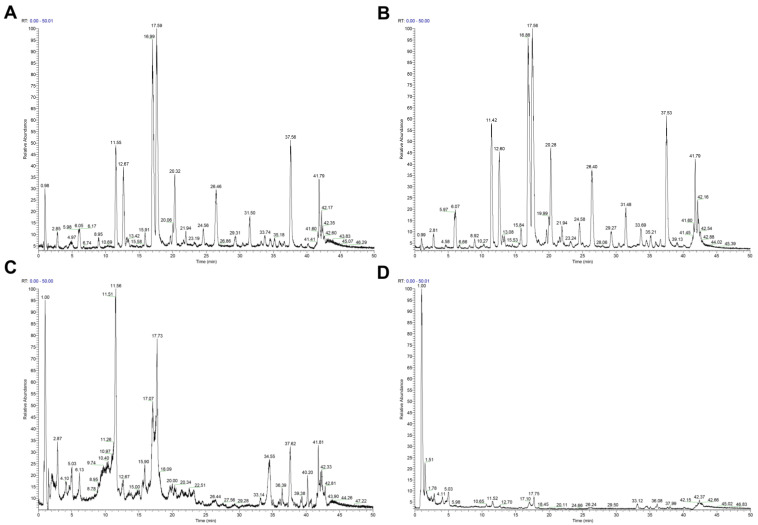
TIC plots of each extract of Xanthocerais lignum in negative ionization mode: (**A**) ethanol extract; (**B**) ethyl acetate fraction; (**C**) n-butanol fraction; (**D**) water fraction.

**Figure 2:**
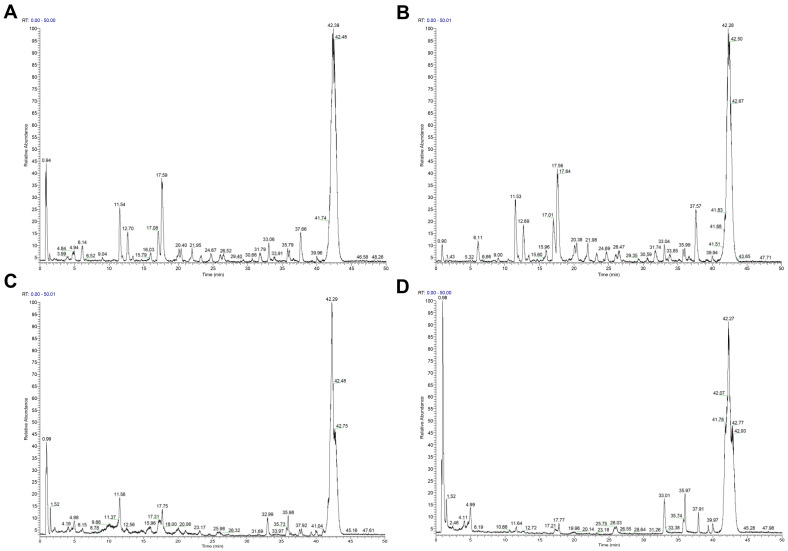
TIC plots of each extract of Xanthocerais lignum in positive ionization mode: (**A**) ethanol extract; (**B**) ethyl acetate fraction; (**C**) n-butanol fraction; (**D**) water fraction.

**Figure 3:**
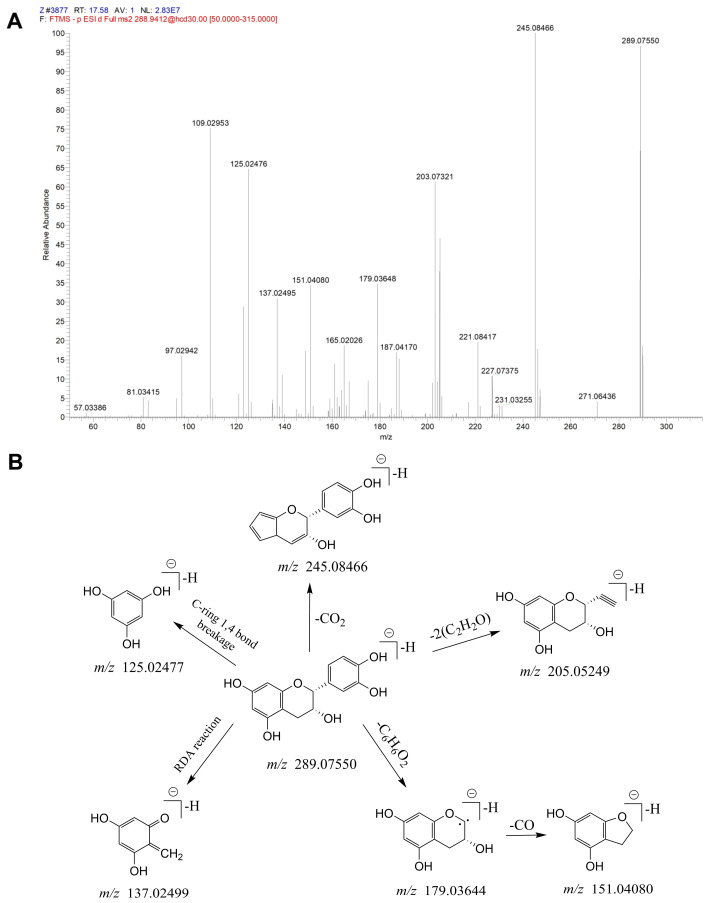
Mass spectrometric identification of epicatechin: (**A**) MS/MS plot of epicatechin; (**B**) mass spectral fragmentation patterns of epicatechin.

**Figure 4:**
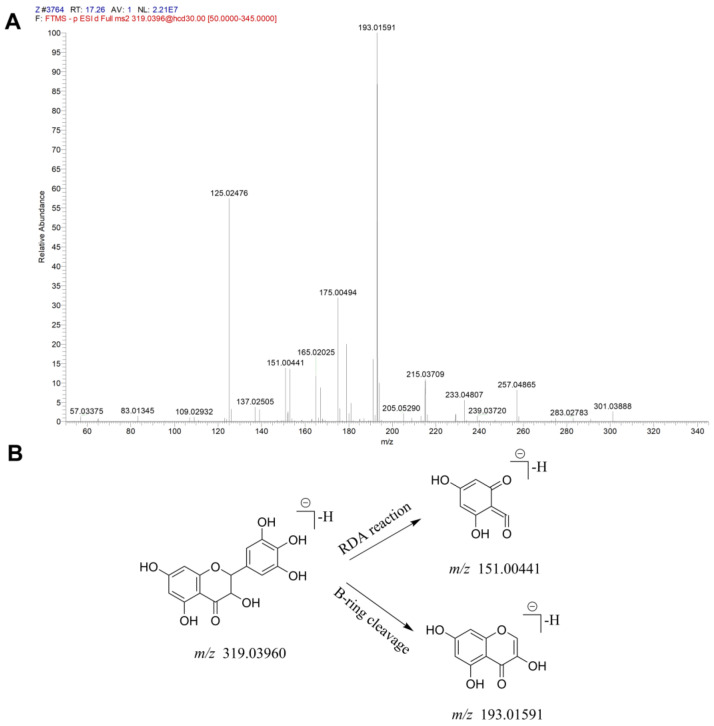
Mass spectrometric identification of dihydromyricetin: (**A**) MS/MS plot of dihydromyricetin; (**B**) mass spectral fragmentation patterns of dihydromyricetin.

**Figure 5:**
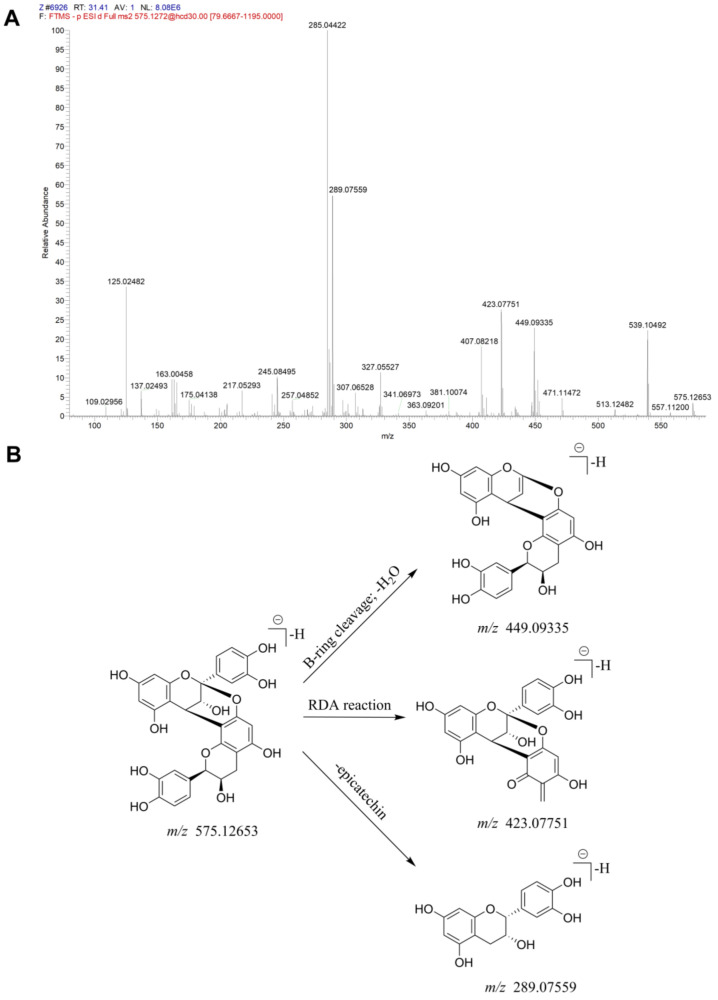
Mass spectrometric identification of proanthocyanidin A2: (**A**) MS/MS plot of proanthocyanidin A2; (**B**) mass spectral fragmentation patterns of proanthocyanidin A2.

The total ion current (TIC) graphs of the various extracts of Xanthocerais lignum in negative and positive ion modes are shown in Figure 1 and Figure 2, respectively. A comparison reveals that there are more chromatographic peaks and better chromatographic separation and signal strength in the negative ion mode. The ethanolic total extract and ethyl acetate fraction of Xanthocerais lignum exhibited a higher similarity in terms of their TIC profiles. Furthermore, these fractions demonstrated a significantly higher number of chromatographic peaks and superior peak shapes compared to the n-butanol fraction and water fraction. UPLC-Q-Exactive Orbitrap MS analysis identified a total of 71 compounds from the various extracts of Xanthocerais lignum, including 48 in the ethanol total extract, 46 in the ethyl acetate fraction, 45 in the n-butanol fraction, and 35 in the water fraction. Detailed information on the identification of chemical constituents is shown in Table 1. The characteristic components of Xanthocerais lignum are flavan-3-ol and flavanone compounds, in addition to proanthocyanidins formed by the polymerization of flavan-3-ol. The mass spectral fragmentation patterns of compounds such as epicatechin, dihydromyricetin, and proanthocyanidin A2, which have the highest relative content in their respective compound classes, are used to illustrate the mass spectral fragmentation patterns of that class of compounds, with specific fragmentation pathways shown in Figure 3, Figure 4 and Figure 5. Epicatechin loses one molecule of CO_2_, resulting in a fragment ion at *m*/*z* 245. It also loses two molecules of C_2_H_2_O, producing a fragment ion at *m*/*z* 205. Additionally, it undergoes B-ring cleavage, generating a fragment ion at *m*/*z* 179. Furthermore, on the basis of the 179-fragment ion, it loses one molecule of CO, resulting in a fragment ion at *m*/*z* 151. Catechin also undergoes retro-Diels-Alder (RDA) reaction, leading to a fragment ion at *m*/*z* 137. Moreover, C-ring 1,4 bond breakage produces a fragment ion at *m*/*z* 125; Dihydromyricetin primarily undergoes B-ring cleavage and RDA cleavage, producing fragment ions at *m*/*z* 193 and *m*/*z* 151, respectively; Proanthocyanidin A2, similarly, mainly undergoes B-ring cleavage and RDA cleavage, resulting in fragment ions at *m*/*z* 449 and *m*/*z* 423, respectively. Additionally, it can lose one molecule of epicatechin, generating a fragment ion at *m*/*z* 289.

### 2.2. Optimal Blood Sampling Time

**Figure 6:**
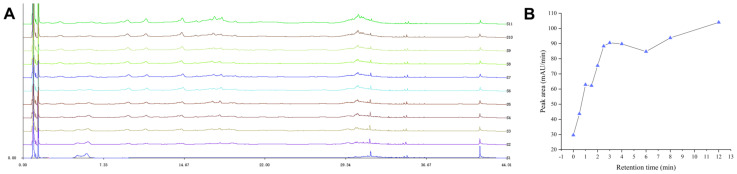
Optimization of blood collection time after the administration of Xanthocerais lignum: (**A**) Chromatograms at each blood collection time point (S1–S11: before administration, 0.5, 1, 1.5, 2, 2.5, 3, 4, 6, 8, and 12 h after administration); (**B**) Total chromatographic peak area at each blood collection time point.

The chromatograms of each blood sampling time point are shown in Figure 6A, while the total peak areas at each time point are presented in Figure 6B. By comparing the chromatograms at different time points, it was observed that at 3 h after administration, the chromatographic peaks exhibited excellent peak shape and separation, and the highest number of chemical components were absorbed into the bloodstream (highest number of chromatographic peaks). Additionally, this time point represented the first peak of drug absorption after administration (the first peak in Figure 6B). To accurately identify more components of Xanthocerais lignum in the bloodstream, the time point of 3 h after administration was ultimately selected as the optimal blood sampling time.

### 2.3. Constituents Absorbed into Blood from Xanthocerais lignum

**Figure 7:**
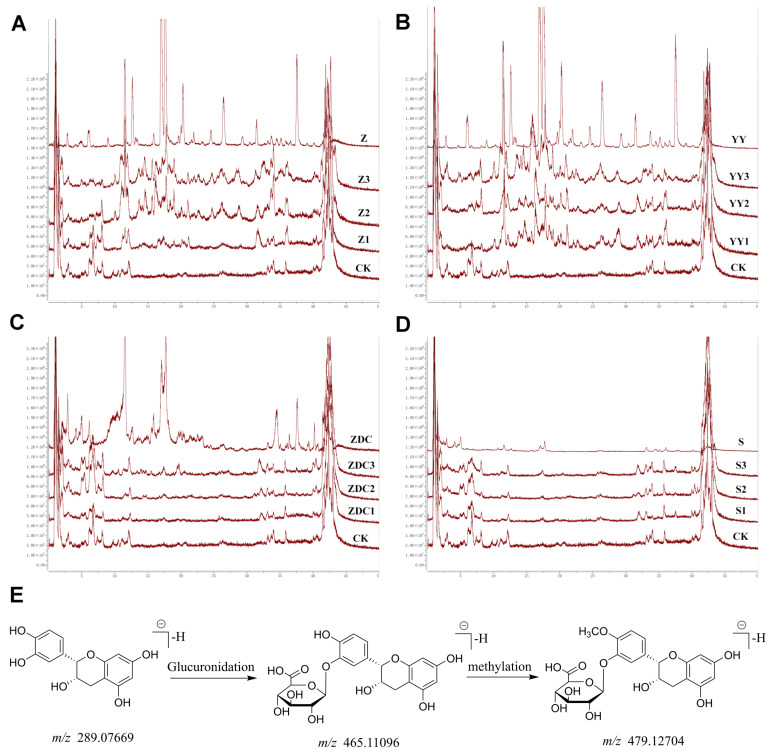
Serum TIC plots of rats with compound metabolic pathways: (**A**) serum TIC plots of rats in the ethanol extract group of Xanthocerais lignum; (**B**) ethyl acetate fraction group; (**C**) n-butanol fraction group; (**D**) water fraction group; (**E**) possible in vivo metabolic pathways of epicatechin.

Figure 7A–D illustrates the comparison of TIC between blank control rat serum, serum from rats treated with various Xanthocerais lignum extracts (ethanol, ethyl acetate, n-butanol, and water), and different classes of Xanthocerais lignum extracts. It can be observed that the TIC of the rat serum after administration shows a significant increase in the number of chromatographic peaks compared to the blank control rat. Among them, the ethyl acetate fraction exhibits the highest number of chromatographic peaks. A total of 36 constituents absorbed into blood were identified, with 24, 27, 14, and 8 components detected in the serum of rats treated with the ethanol total extract, ethyl acetate fraction, n-butanol fraction, and water fraction, respectively. Among these, 17 components corresponded to prototype compounds directly entering the bloodstream, while the remaining 19 components were metabolites. Detailed information on the identification of constituents absorbed into the blood from Xanthocerais lignum is provided in Table 2, and peak areas are listed in Table S1. The results indicate that the metabolic pathways of Xanthocerais lignum’s chemical constituents predominantly involve glucuronidation. Characteristic metabolites identified include flavonoid glucuronides such as epicatechin-3′-*O*-glucuronide and 4′-*O*-methyl-epicatechin-3′-*O*-glucuronide. By correlating the referenced literature [10,11] with mass spectrometry data, the metabolic trajectory of epicatechin within the body is postulated, as depicted in Figure 7E. Epicatechin undergoes glucuronidation to form epicatechin-3′-*O*-glucuronide, followed by methylation to further generate 4′-*O*-methyl-epicatechin-3′-*O*-glucuronide.

### 2.4. Results of Serum Spectrum–Effect Correlation Analysis

The results of the serum spectrum–efficacy correlation analysis are shown in Table 3. The gray correlation values range between 0 and 1, with higher values indicating stronger correlations with the parent sequence (Y) [12], i.e., the stronger the relationship between the compound and the anti-RA efficacy. Among the top 10 compounds, 7 are prototype blood-entering components of Xanthocerais lignum, while the remaining 3 are metabolites resulting from glucuronidation of flavonoids found in Xanthocerais lignum. These compounds exhibit gray correlation values above 0.8, suggesting their potential for effective anti-RA activity.

### 2.5. Mechanism of Xanthocerais lignum’s Anti-RA Q-Markers

**Figure 8:**
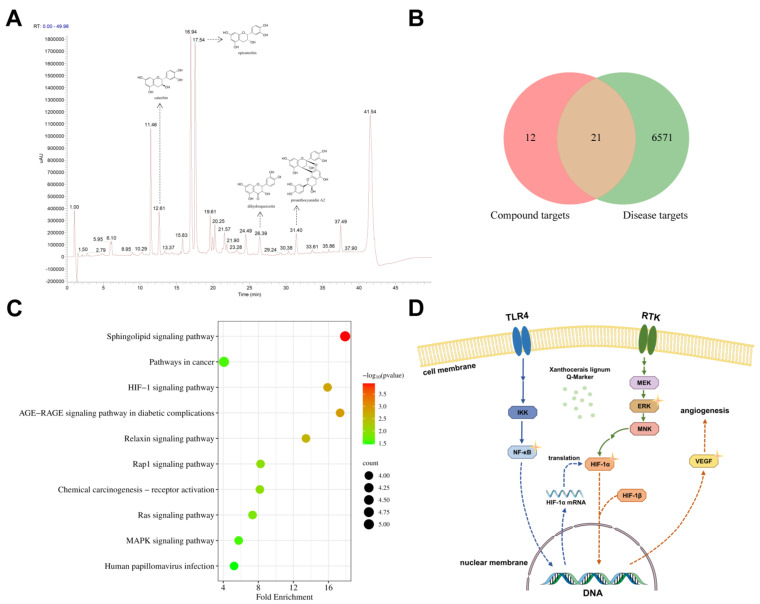
Identification of Q-Marker for anti-RA in Xanthocerais lignum and their mechanisms of action: (**A**) chromatogram of the total extract of Xanthocerais lignum; (**B**) intersection of Q-Marker targets with RA disease targets; (**C**) results of KEGG enrichment analysis; (**D**) possible mechanism of anti-RA action of Q-Marker.

Following the “principles for determining Q-Markers of Chinese medicine” [8], 4 components were selected as Xanthocerais lignum’s anti-RA Q-markers: proanthocyanidin A2, epicatechin, dihydroquercetin, and catechin. These compounds are characteristic chemical constituents of Xanthocerais lignum and exhibit relatively high content. The serum spectrum–effect correlation analysis results also suggest that these 4 compounds may possess potential anti-RA activity. All 4 compounds were further identified by comparison with the standards, and the chromatogram of the Xanthocerais lignum total extract at 230 nm is shown in Figure 8A.

From the ChEMBL database, we have collated 11 experimentally active targets for Q-Marker, with detailed information presented in Table S2. Utilizing the SuperPred database, we have predicted 70 potential targets for Q-Marker. By amalgamating the experimentally active targets with the predicted targets and eliminating duplicates, we have identified 33 unique targets. An aggregate of 6592 RA-related targets was gathered from three databases, among which 21 intersect with the Q-Marker’s targets, as depicted in Figure 8B. KEGG enrichment analysis yielded 33 signaling pathways, and the top 10, based on Count values, were selected for visual analysis, the results of which are illustrated in Figure 8C. These signaling pathways are closely associated with the developmental processes of RA. For instance, the HIF-1 signaling pathway can facilitate the formation of osteoclasts, thereby exacerbating inflammatory bone resorption [13]. Aberrant activation of the Ras signaling pathway can lead to the destruction of RA synovial tissue and inflammation [14]. Activation of the MAPK signaling pathway can induce abnormal proliferation and migration of rheumatoid arthritis fibroblast-like synoviocytes (RA-FLS) [15]. Integrating the enrichment analysis parameters with the RA pathogenesis mechanism, we ultimately selected the HIF-1 signaling pathway for subsequent validation. Within the HIF-1 signaling pathway, the Q-Marker of Xanthocerais lignum against RA may exert pharmacological effects such as inhibiting angiogenesis by targeting ERK, NF-κB, HIF-1α, and VEGF, thereby mitigating the progression of RA, with the detailed mechanism of action delineated in Figure 8D.

### 2.6. Molecular Docking Outcomes

**Figure 9:**
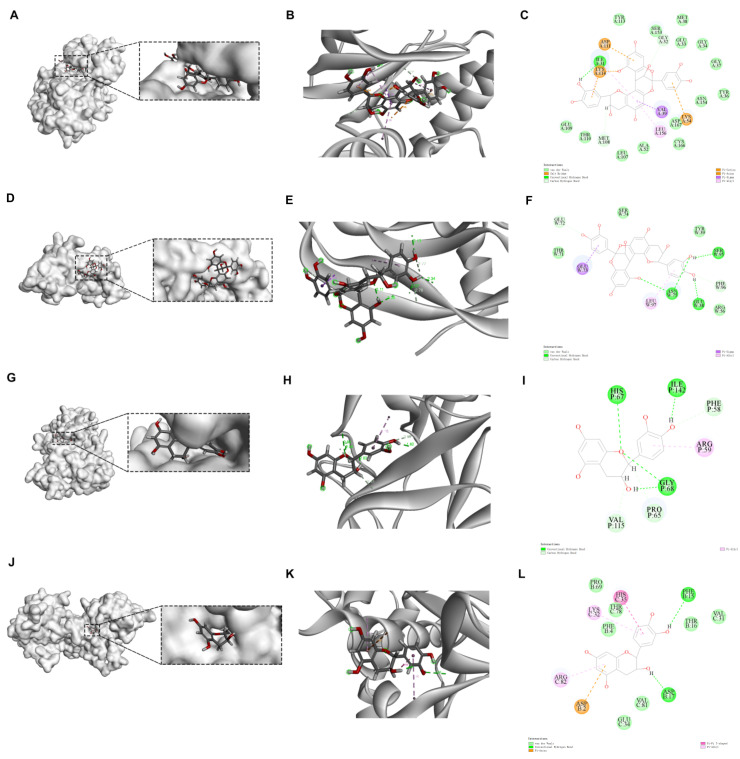
Schematic representation of ligand–protein interactions in molecular docking: (**A**–**C**) procyanidin A2 and ERK; (**D**–**F**) procyanidin A2 and VEGF; (**G**–**I**) epicatechin and NF-κB; (**J**–**L**) Catechin and HIF-1α.

Table 4 delineates the molecular docking results of Xanthocerais lignum’s anti-RA Q-Markers with 4 targets within the HIF-1 signaling pathway. Proanthocyanidin A2 demonstrated the lowest binding energy with ERK and VEGF targets, epicatechin with NF-κB, and catechin with HIF-1α. The ligand–protein interactions of these compounds are illustrated in Figure 9. Proanthocyanidin A2 formed hydrogen bonds at positions 31 (isoleucine), 32 (glycine), and 108 (methionine) within the A-chain of the ERK protein; it also formed 2 hydrogen bonds at positions 75 (asparagine) and 95 (serine), and 1 hydrogen bond at positions 38, 78 (glutamic acid), and 96 (phenylalanine) within the W-chain of the VEGF protein. Epicatechin established 2 hydrogen bonds at positions 68 (glycine) and 1 hydrogen bond at positions 58 (phenylalanine), 65 (proline), 67 (histidine), 115 (valine), and 142 (isoleucine) within the P-chain of the NF-κB protein. Catechin interacted through hydrogen bonds with phenylalanine at position 15 and aspartic acid at position 17 within the B-chain of the HIF-1α protein. In addition to hydrogen bonds, the complexes exhibited π–cation interactions, van der Waals forces, and other non-covalent interactions, which are crucial in determining the stability and binding affinity of the ligand–protein complexes. From the docking results, it can be observed that the ligand–protein complex with the lowest binding affinity forms a minimum of 2 hydrogen bonds. Compared to other non-covalent interactions, hydrogen bonds make a greater contribution to the stability of the complex.

Generally, binding affinity is determined by the free energy of binding, with a stable binding conformation correlating with lower binding energy. A binding energy less than 0 indicates that the ligand can spontaneously bind to the receptor, with smaller values signifying higher binding activity and ease of drug–receptor interaction [16]. The negative binding energies across all ligand–protein interactions suggest a favorable binding affinity, further corroborating the mechanism through which Xanthocerais lignum’s Q-Markers exert anti-RA effects. The active components present in Xanthocerais lignum may potentially inhibit the activity of the aforementioned proteins by forming complexes with them, thereby exerting an anti-RA effect.

## 3. Discussion

The CIA (collagen-induced arthritis) model was initially established in Wistar and Wistar–Lewis rats using type II collagen immunization and was later expanded to include mice and non-human primates [17]. Due to their similar immune and pathological characteristics to RA, these CIA models have been widely employed in human RA research. Typically, the construction of the CIA model in rats requires animals that are at least 7–8 weeks old when their immune systems have matured. Wistar rats are a well-established strain highly susceptible to CIA, and in our study, approximately 70% of the rats successfully developed the CIA model, making them suitable for subsequent screening of Q-Markers from Xanthocerais lignum with potential anti-RA properties.

Differences were observed in the identified compounds among the various extracts of Xanthocerais lignum. The relative content of flavonoid aglycone was significantly higher in the ethanolic total extract and ethyl acetate fraction compared to the n-butanol fraction and water fraction. This disparity may be attributed to the higher extraction efficiency of ethyl acetate during the extraction process, effectively enriching the flavonoids in Xanthocerais lignum. Numerous studies have demonstrated the favorable anti-RA activity of flavonoids such as quercetin and epigallocatechin gallate. The mechanisms underlying their anti-RA effects primarily involve anti-inflammatory, antioxidant, and immunomodulatory actions [18]. The significant anti-RA activity observed in the ethyl acetate fraction is closely associated with its rich content of flavonoids. Flavonoid compounds exhibit greater selectivity and sensitivity in negative ion mode during LC–MS analysis [19], resulting in superior chromatographic separation and signal intensity in the TIC chart when using negative ion mode.

The total ethanol extract of Xanthocerais lignum showed an in vivo absorption trend that initially increased, then decreased, and subsequently increased again over time. This trend may be attributed to the complex chemical composition of the traditional Chinese medicine extract, which could involve multiple drug interactions affecting absorption, distribution, metabolism, and excretion within the body. Moreover, different chemical components may exhibit distinct pharmacokinetic characteristics [20]. For instance, some components may be rapidly absorbed but also quickly metabolized and excreted, leading to an initial increase followed by a decrease in their exposure levels within the body. Conversely, other components may be absorbed more slowly, yet their metabolism and excretion are also slow, resulting in a later increase in exposure levels. Due to the complexity of herbal ingredients, studying their pharmacokinetics poses significant challenges. Currently, a widely accepted approach is to represent the overall pharmacokinetic behavior of herbal medicine by investigating the pharmacokinetic characteristics of one or several known active constituents [21]. For example, the pharmacokinetics of 2*α*-hydroxyl-3*β*-angeloylcinnamolide can be studied to represent the pharmacokinetic profile of *Polygonum jucundum*, while the pharmacokinetic characteristics of 2,3,5,4′-tetrahydroxystilbene-2-*O*-*β*-D-glucoside can be used to associate with *Polygonum multiflorum* [22,23]. Our research indicates that the compounds proanthocyanidin A2, epicatechin, dihydroquercetin, and catechin exhibit potential anti-RA activity. Furthermore, all 4 compounds can be quantitatively analyzed in both the blood of rats and the extracts of Xanthocerais lignum using UPLC. Thus, the pharmacokinetic characteristics of Xanthocerais lignum can be characterized by conducting pharmacokinetic studies on one or more of these compounds.

Multiple constituents of Xanthocerais lignum can be absorbed by rats and enter the bloodstream, with the prototype blood-entering components being organic acids and flavonoid compounds. Some of these are metabolic products within the organism or substances involved in metabolism, such as uric acid, an end product of purine metabolism that serves as an antioxidant protecting cells from free radical damage [24]. Citric acid is a crucial intermediate in the tricarboxylic acid cycle, participating in energy metabolism and biosynthesis [25]. Methylmalonic acid, an intermediate product of branched-chain amino acid metabolism, can be converted into succinyl-CoA by methylmalonyl-CoA mutase, entering the tricarboxylic acid cycle [26]. These compounds are involved in metabolic processes that are widespread in both animals and plants; hence, they can be detected in both Xanthocerais lignum and rat serum. The remaining prototype blood-entering components are secondary metabolites of plants, primarily flavan-3-ol and flavanone compounds, which are relatively abundant in Xanthocerais lignum and possess various pharmacological activities such as antioxidant, anti-inflammatory, and antitumor effects [27].

Some of the flavonoid components in Xanthocerais lignum are converted to flavonoid glucuronides in vivo. Glucuronidation is an important phase II metabolic reaction catalyzed by glucuronosyltransferases, responsible for the clearance of various endogenous and exogenous substances within the body, significantly influencing the efficacy and adverse reactions of drugs [28]. Most drugs become more water-soluble and have less pharmacological activity after glucuronidation. Studies have shown that the glucuronidated products of (−)-epicatechin exhibit reduced antioxidant activity compared to the original components [29].

In the serum spectrum–effect correlation analysis, the top 10 ranked compounds are predominantly plant flavonoids with antioxidant, anti-inflammatory, and immunomodulatory effects. They can inhibit the production of RA-related cytokines and inflammatory mediators, such as TNF-*α* and IL-6, and reduce joint inflammation and damage by suppressing synovial cell proliferation and migration, as well as osteoclast differentiation [30]. Additionally, in vitro and in vivo experiments have confirmed the efficacy of these compounds against RA. For example, procyanidin A2 can target the NF-κB, MAPK, and Nrf2 pathways to exert anti-inflammatory and antioxidant effects [31]. Catechins can reduce secondary inflammation in rats by inhibiting the production of IL-1, TNF-*α*, and PGE2 in an adjuvant-induced arthritis model and by upregulating the expression of EP2 in rat synovial cells [32]. Epicatechin significantly inhibits the activation of the NLRP3 inflammasome and the NF-κB signaling pathway by inflammatory cytokines (IL-1*β*, IL-18, IL-6, and TNF-*α*) both in vitro and in vivo, thereby exerting anti-inflammatory effects [33]. Dihydroquercetin can inhibit RANKL-induced osteoclast differentiation and gene expression, including TRAP and MMP-9, showing potential for treating osteoporosis, bone resorption, and related diseases such as RA [34].

Based on the core theory of “Chinese medicine efficacy—pharmacodynamic material basis—quality control characteristic components”, 4 Q-Markers of Xanthocerais lignum have been identified. These markers satisfy the unique specificity, measurability, and effectiveness principles of Q-Markers and can serve as quality indicators for Xanthocerais lignum’s anti-RA properties. The relative contents of the 4 Q-Markers in Xanthocerais lignum can be used to preliminarily evaluate the strength of its anti-RA activity. HIF-1 is a heterodimeric transcription factor composed of HIF-1α and HIF-1β subunits, primarily responsive to changes in oxygen concentration. In RA, the hypoxic microenvironment of the joints is the main inducer of HIF-1 [35]. HIF-1 can exacerbate the RA process by promoting inflammation, angiogenesis, and cartilage destruction [36], with the regulation of VEGF gene expression by HIF-1α being a key factor in promoting angiogenesis [37]. HIF-1α is regulated by various signaling pathways, and inhibiting the activation of the MAPK and NF-κB signaling pathways can reduce the expression of HIF-1α, thereby alleviating the RA process [36,38]. Existing research suggests that elevated expression of HIF-1α facilitates the migration and invasion of RA-FLS, thereby exacerbating the erosion of surrounding cartilage [39]. It is postulated that the 4 Q-Markers found in Xanthocerais lignum may exert anti-RA effects by inhibiting the activation of the HIF-1 signaling pathway.

## 4. Materials and Methods

### 4.1. Experimental Animals

Female Wistar rats, aged 7–8 weeks, weighing 200–220 g, totaling 20 individuals, were procured from SiPeiFu (Beijing) Biotechnology Co., Ltd. (Beijing, China), with license number SCXK (Beijing) 2019-0010. The experimental animals were housed in an SPF environment with unrestricted access to water and food. All animal experiments in this study were approved by the Medical Ethics Committee of Inner Mongolia Medical University (approval number: YKD202301197) and adhered to internationally accepted principles for laboratory animal use and care.

### 4.2. Drugs and Reagents

Xanthocerais lignum medicinal materials (AnGuo RunDe Pharmaceutical Co., Ltd. (AnGuo, China), batch number: C20112813), authenticated by Professor Qu Bi of Inner Mongolia Medical University as the dried stem or branch wood of *Xanthoceras sorbifolia* Bunge., are currently preserved in the specimen room of Inner Mongolia Medical University. The standards used include catechin, epicatechin, dihydroquercetin, and proanthocyanidin A2 (purity ≥ 98%, HERBPURIFY Biotechnology Co., Ltd. (Chengdu, China), batch numbers: E-01111812016, B02011812016, E-00111812016, and Y-16511812016). The solvents used include anhydrous ethanol and ethyl acetate (analytical grade, Tianjin JinDongTianZheng Chemical Reagent Factory (Tianjin, China), batch numbers: 20211008 and 20180720), n-butanol (analytical grade, Tianjin FengChuan Chemical Reagent Co., Ltd. (Tianjin, China), batch number: 20210523), and methanol and acetonitrile (chromatographic grade, Fisher Scientific, Waltham, MA, USA, batch numbers: L-14734 and L-14834).

### 4.3. Main Instruments

JYT-50LC ultrasonic extraction and concentration device (Shanghai JuYuan Automation Technology Co., Ltd., Shanghai, China); EYELA-N1300 rotary evaporator, FDU-2110 freeze dryer, UT-2000 centrifugal concentrator (EYELA, Tokyo, Japan); bluepard V2 vortexer (Shanghai YiHeng Scientific Instrument Co., Ltd., Shanghai, China); UltiMate 3000 high-performance liquid chromatograph, UPLC-Q-Exactive Orbitrap MS (Thermo Fisher Scientific, Waltham, MA, USA).

### 4.4. Preparation of Various Extracts of Xanthocerais lignum

#### 4.4.1. Preparation of Total Ethanol Extract

The dried Xanthocerais lignum medicinal material, weighing 1 kg, is pulverized and passed through a number 4 sieve (Sieve inner diameter: 250 μm ± 9.9 μm). Subsequently, the powdered material is added to an 8 L solution of 70% ethanol. Using an ultrasonic extraction and concentration device, the material is extracted 3 times at a temperature of 80 °C, with a power of 250 W and a frequency of 40 kHz, with each extraction lasting 2 h. The extracted liquids are combined, and the alcoholic content is removed through vacuum concentration using a rotary evaporator until no alcohol odor remains. The resulting extract is then subjected to freeze-drying, yielding a powdered form of the ethanol extract of Xanthocerais lignum.

#### 4.4.2. Preparation of Different Polarity Extracts

The aforementioned total ethanol extract of Xanthocerais lignum (Z) was suspended in water and then successively extracted with 1.5 volumes of ethyl acetate and n-butanol solutions, 3 times each. The respective extracts were combined, and the different polarity extracts and water fractions were concentrated under reduced pressure and then freeze-dried to obtain freeze-dried powders of the ethyl acetate fraction (YY), n-butanol fraction (ZDC), and water fraction (S) of Xanthocerais lignum.

### 4.5. Study of Chemical Constituents of Xanthocerais lignum

#### 4.5.1. Preparation of Test Sample Solutions

Precisely weigh the freeze-dried powders of the total ethanol extract, ethyl acetate fraction, n-butanol fraction, and water fraction of Xanthocerais lignum to 0.1001 g, 0.1035 g, 0.1005 g, and 0.1004 g, respectively. Dissolve each powder in chromatographic methanol and make up 10 mL in a volumetric flask. Centrifuge each solution at 16,000 r·min^−1^ for 10 min at 4 °C and filter the supernatant through a 0.22 μm microporous membrane to obtain the test sample solution.

#### 4.5.2. Chromatographic and Mass Spectrometric Conditions

Chromatographic conditions: Chromatographic column: Syncronis C_18_ liquid chromatography column (100 mm × 2.1 mm, 1.7 μm); mobile phase: acetonitrile (A)~0.4% acetic acid water (B); elution gradient: 0~18 min, 5%~15% A; 18~25 min, 15% A; 25~38 min, 15%~25% A; 38~39 min, 25%~100% A; 39~40 min, 100%~5% A; injection volume: 3 µL for the total ethanol extract and ethyl acetate fraction of Xanthocerais lignum, and 10 µL for the n-butanol fraction and water fraction; column temperature: 20 °C; flow rate: 0.3 mL·min^−1^.

Mass spectrometric conditions: Mass spectrometry was performed using an ESI source in both positive and negative ion modes, with the following parameters for each mode: ion source voltage 4 kV(+)/3.2 kV(−); sheath gas flow rate 40 L·min^−1^(+)/35 L·min^−1^(−); fragmentation voltage 300 V; drying gas temperature 350 °C; auxiliary gas flow rate 2 L·min^−1^; spray gas pressure 45 psig; high-purity nitrogen as the nebulizing gas; data acquisition range 100~1100 *m*/*z*, using full MS-ddMS2 scanning mode.

#### 4.5.3. Identification of Chemical Constituents

Qualitative analysis of the test sample solutions was performed using UPLC-Q-Exactive Orbitrap MS technology. Initially, a compound library of Xanthocerais lignum was established based on existing literature reports (relative molecular mass and secondary mass spectral information were obtained from PubChem and MassBank, respectively). The primary and secondary mass spectral information for each chromatographic peak was searched and matched by combining the in-house library in Compound Discoverer 3.1 (includes compound name, relative molecular mass, and secondary mass spectral information) with the Xanthocerais lignum compound library. For chromatographic peaks without matching data, the molecular formula of each peak was preliminarily determined based on the actual measured relative molecular mass and the theoretical exact relative molecular mass. The compounds were searched in MassBank (www.massbank.jp, accessed on 5 April 2024) and mzCloud (www.mzcloud.org, accessed on 5 April 2024) databases according to their molecular formula or relative molecular mass. The primary and secondary mass spectral fragmentation data of the chromatographic peak were compared with the compounds in the databases, and the chemical structures were identified in conjunction with the mass spectral fragmentation patterns of this class of compounds.

### 4.6. Animal Modeling and Grouping

After 1 week of acclimatization, a CIA rat model was constructed. The modeling procedure involved dissolving bovine type II collagen in 0.05 mol·L^−1^ glacial acetic acid, resulting in a final concentration of 2 mg·mL^−1^. The dissolved collagen was then refrigerated overnight at 4 °C. The following day, it was mixed with an equidose of Freund’s incomplete adjuvant and thoroughly emulsified at 4 °C. The CIA rat model was established by subcutaneously injecting a total volume of 0.2 mL of the collagen emulsion into the base of the tail and the footpad. Immune enhancement was achieved after 1 week by injecting an additional 0.1 mL of the collagen emulsion using the same method. After 3 weeks of initial immunization, 14 out of the 20 rats exhibited complete redness and swelling in all of their paws, including the ankle joints, signifying successful modeling. From this group of rats, 12 rats were randomly selected and assigned to different treatment groups: the total ethanol extract of Xanthocerais lignum (Z), ethyl acetate fraction (YY), n-butanol fraction (ZDC), and water fraction (S). Each group consisted of 3 rats, while the remaining 2 rats were used as blank controls and for optimizing the blood collection time, respectively.

### 4.7. Serum Medicinal Chemistry Studies of Xanthocerais lignum

#### 4.7.1. Preparation of Serum Test Sample Solutions

All rats were fasted for 12 h but allowed access to water before oral administration of the drug. The freeze-dried powders of the ethanol extract of Xanthocerais lignum, ethyl acetate fraction, n-butanol fraction, and water fraction were suspended in a 0.5% CMC-Na solution. The administration was conducted according to the designated groups, with a dosage of 1.35 g·kg^−1^ for each group (human clinical dosage was 3 g·d^−1^, which was 5 times the human clinical daily dose based on body surface area). Administration continued for 3 consecutive days to enhance the response of the blood-entering components of Xanthocerais lignum. The blank control rats received an equivalent volume of CMC-Na solution for 3 consecutive days. After the final administration, approximately 0.6 mL of blood was collected from each rat via the jugular vein. The collected blood was allowed to rest at room temperature for 1 h and then centrifuged at 5000 rpm for 10 min at 4 °C. A 200 µL aliquot of the supernatant was mixed with 800 µL of methanol and vortexed for 30 s to precipitate proteins. The mixture was further centrifuged at 15,000 rpm for 10 min at 4 °C to separate the proteins. The supernatant was collected and evaporated using a centrifugal concentrator. Subsequently, 200 µL of methanol was added, followed by vortexing for 2 min. The mixture was then centrifuged at 16,000 rpm for 10 min at 4 °C, and the supernatant was filtered through a 0.22 µm microporous membrane to obtain the serum test solution.

#### 4.7.2. Chromatographic and Mass Spectrometric Conditions

As per Section 4.5.2, the sample volume for each group of rat serum test samples was 10 µL.

#### 4.7.3. Optimization of Optimal Blood Collection Time

Blood was collected before administration and at 0.5, 1, 1.5, 2, 2.5, 3, 4, 6, 8, and 12 h after administration of Xanthocerais lignum ethanol total extract. The serum test sample solution was prepared according to the method in Section 4.7.1. The optimal blood sampling time is determined based on the number of chromatographic peaks, peak areas, and separation degree at each time point.

#### 4.7.4. Identification of Prototype Blood-Entering Components and Metabolites

The serum test sample solution was prepared according to the method in Section 4.7.1 (blood collected at the optimal time point), and UPLC-Q-Exactive Orbitrap MS technology was used for detection. Compound identification is performed according to the method outlined in Section 4.5.3. By comparing the blood components of the blank control rats with those of the medicated group rats, combined with the chemical components of Xanthocerais lignum, the prototype blood-entering components and metabolites of Xanthocerais lignum are clarified.

### 4.8. Serum Spectrum–Effect Correlation Analysis of Xanthocerais lignum

**Figure 10:**
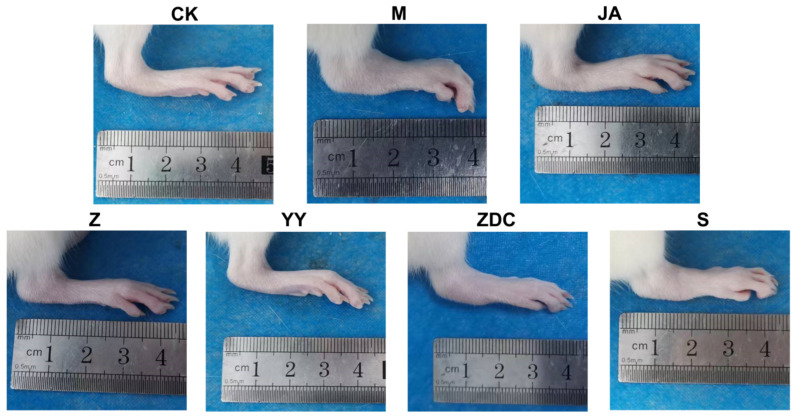
Therapeutic effect of each administration group on CIA rats (CK: blank control group; M: CIA model group; JA: methotrexate group).

Our research group previously evaluated the anti-RA effects of various extracts of Xanthocerais lignum through multiple methods, such as joint swelling measurement, tissue pathology observation, and cytokine level detection, from holistic, organ-tissue, and molecular levels. The therapeutic effect of each administration group on arthritis in rats is shown in Figure 10. These pharmacological data are reverse-normalized (after mapping the data uniformly to the range [0, 1], the values are subtracted from 1 to ensure that the pharmacological information and the therapeutic efficacy of the drugs are positively correlated) and assigned different weights (0.4 × holistic level efficacy + 0.3 × organ-tissue level efficacy + 0.4 × molecular level efficacy) as dependent variables (Y) in the spectrum–effect correlation analysis. The peak areas of the constituents absorbed into the blood from various extracts of Xanthocerais lignum were used as independent variables (X) for grey relational analysis. The anti-RA pharmacological information of various extracts of Xanthocerais lignum is shown in Table S3. During the analysis, mean normalization was used as a dimensionless treatment method (data of the parent sequence and the characteristic sequence were divided by their respective mean value), with a resolution coefficient *ρ* = 0.5 (the smaller the resolution coefficient is, the larger the resolution is, and the best resolution is achieved at *ρ* ≤ 0.5463, usually set at *ρ* = 0.5). The gray correlation values are obtained by solving the gray correlation coefficient between the parent sequence (Y) and the characteristic sequence (X), and the pharmacodynamic material basis of Xanthocerais lignum against RA was determined based on the ranking results of the gray correlation values.

### 4.9. Prediction of the Mechanism of Q-Markers of Xanthocerais lignum against RA

After determining the pharmacodynamic material basis of Xanthocerais lignum against RA, the Q-Markers of Xanthocerais lignum against RA were screened based on the “principles for determining Q-Markers of Chinese medicine” [8], and then compared with standards. The screening criteria are as follows: (1) Effectiveness: compounds ranked in the top 10 based on serum spectrum–effect correlation analysis. (2) Measurability: compounds detectable in both Xanthocerais lignum herbal material and rat serum. (3) Specificity: characteristic chemical components in Xanthocerais lignum, primarily belonging to the flavanols and flavanone classes.

Experimental activity targets of Q-Markers were collected from the ChEMBL database (www.ebi.ac.uk/chembl, accessed on 21 April 2024), and potential action targets of Q-Markers were predicted using the SuperPred database (https://prediction.charite.de, accessed on 21 April 2024), with a selection criterion of Probability > 80%. The Gene ID Conversion Tool in the DAVID database (https://david.ncifcrf.gov, accessed on 21 April 2024) was used to convert “Uniprot ID” to “Official Gene Symbols”.

RA-related targets were collected from the Gene Cards (www.genecards.org, accessed on 21 April 2024), DisGeNET (www.disgenet.org/home, accessed on 21 April 2024), and OMIM (www.omim.org, accessed on 21 April 2024) databases. After intersecting the disease targets with the Q-Marker targets, the overlapping targets were imported into the DAVID database for KEGG enrichment analysis. The *p*-values were corrected using the false discovery rate (FDR) control method (FDR < 0.05). Ultimately, significant signaling pathways were selected based on a threshold of *P* < 0.05 to predict potential action pathways and targets of Q-Markers in the context of RA treatment with Xanthocerais lignum.

### 4.10. Validation by Molecular

Docking Protein receptor structures were obtained from the PDB database (www.rcsb.org, accessed on 22 April 2024), and small molecule ligand structures were obtained from the PubChem database (https://pubchem.ncbi.nlm.nih.gov, accessed on 22 April 2024). After processing the receptor and ligand molecules using Discovery Studio 2019, molecular docking was performed using the CDOCKER method. For protein receptor complexes with inherent small molecule ligands, binding sites were generated at the location of the ligand molecule; if the protein receptor did not contain a small molecule ligand, binding sites were sought within the receptor cavity. After docking, the CDOCKER interaction energy was calculated, and the compound with lower binding energy was selected for analysis of its ligand–protein interactions.

## 5. Conclusions

In this study, 15 flavonoid compounds were identified from Xanthocerais lignum, primarily belonging to the classes of flavanols and dihydroflavones. Among them, 7 compounds were observed to be absorbed into the bloodstream in their original forms, while 4 compounds underwent metabolism via the glucuronidation pathway. Serum spectrum–effect correlation analysis results suggest that procyanidin A2, dihydroquercetin, catechin, epicatechin, and their glucuronidated products may possess potential anti-RA activity, serving as the pharmacodynamic material basis for Xanthocerais lignum’s anti-RA effects. Based on these findings, 4 components were selected as Q-Markers for Xanthocerais lignum’s anti-RA properties. The action mechanisms of these components, as predicted using compound–target interaction data and molecular docking techniques, may involve modulating the expression of proteins such as ERK, NF-κB, HIF-1α, and VEGF within the HIF-1 signaling pathway. This modulation may exert pharmacological effects that inhibit inflammation, angiogenesis, and cartilage destruction, thereby alleviating the progression of RA. However, the detailed mechanisms of action require further validation through in vitro and in vivo experiments. For instance, in vitro assessments can be conducted using human or animal cell lines pertinent to RA to examine the impact of Q-Markers on key proteins within the HIF-1 signaling pathway, such as ERK, NF-κB, HIF-1α, and VEGF. Furthermore, in vivo studies utilizing CIA models can be conducted to observe the therapeutic effects of the Q-Markers, including the evaluation of inflammatory cytokines, angiogenesis, and joint pathological changes. Xanthocerais lignum has broad application prospects for anti-RA treatment. This study provides valuable references for the quality assessment of Xanthocerais lignum and the further development of lead compounds with anti-RA activity derived from Xanthocerais lignum.

## Figures and Tables

**Table 1:** Chemical constituents of each extract of Xanthocerais lignum.

No	t_R_/min	Molecular Formula	Actual Measured *M*_r._	Theoretical Exact *M*_r._	Secondary Mass Spectral Fragmentation	Compounds	Source
1	0.955	C_12_H_22_O_11_	387.11990 [M + HCOO]^−^	342.11621	341.11288, 179.05743, 119.03507, 113.02441, 89.02404	*α*-lactose	Z
2	0.982	C_5_H_12_O_5_	151.06256 [M − H]^−^	152.06847	119.03532, 101.02454, 89.02424, 71.01341, 59.01327	L-(−)-arabitol	Z, ZDC, S
3	0.991	C_12_H_22_O_11_	341.11420 [M − H]^−^	342.11621	179.05785, 119.03532, 101.02451, 89.02422, 59.01324	*α*,*α*-trehalose	S
4	1.013	C_6_H_6_N_4_O_2_	165.04208 [M − H]^−^	166.04908	129.01997, 75.00842	7-methylxanthine	Z, ZDC, S
5	1.022	C_5_H_10_O_5_	149.04680 [M − H]^−^	150.05282	101.02453, 89.02424, 71.01340, 59.01326	D-(−)-ribose	Z, ZDC, S
6	1.027	C_8_H_14_O_7_	221.06966 [M − H]^−^	222.07395	129.02008, 85.02930, 72.99276, 59.01329	ethyl-*β*-d-glucuronide	S
7	1.039	C_4_H_6_O_5_	133.01527 [M − H]^−^	134.02152	115.00395, 89.02425, 71.01343, 59.01318	DL-malic acid	Z, YY, ZDC, S
8	1.042	C_6_H_8_O_7_	191.02213 [M − H]^−^	192.02700	173.01054, 129.02000, 111.00899, 87.00854, 85.02927, 57.03399	citric acid	Z, ZDC, S
9	1.062	C_4_H_8_O_5_	135.03085 [M − H]^−^	136.03717	117.01963, 75.00843	L-threonic acid	Z, ZDC, S
10	1.082	C_6_H_12_O_6_	179.05827 [M − H]^−^	180.06339	119.03536, 113.02467, 101.02457, 89.02426, 71.01343, 59.01328	D-(+)-glucose	Z, YY, ZDC, S
11	1.083	C_6_H_14_O_6_	181.07390 [M − H]^−^	182.07904	163.06238, 119.03535, 101.02454, 89.02425, 71.01343, 59.01328	D-(−)-mannitol	Z, YY, ZDC, S
12	1.099	C_6_H_10_O_5_	161.04715 [M − H]^−^	162.05282	101.02460, 99.04536, 59.01328, 57.03459	3-hydroxy-3-methylglutaric acid	Z, ZDC, S
13	1.522	C_5_H_7_NO_3_	128.03622 [M − H]^−^	129.04259	84.04505, 82.02949	4-oxoproline	Z, ZDC, S
14	1.524	C_5_H_5_N_5_	134.04825 [M − H]^−^	135.05450	107.03640	adenine	ZDC
15	1.526	C_5_H_5_N_5_O	150.04346 [M − H]^−^	151.05073	133,01624, 108.02042, 107.03648, 82.04082, 78.00912, 66.00903	guanine	ZDC
16	1.527	C_5_H_4_N_4_O_3_	167.02277 [M − H]^−^	168.02834	125.01711, 124.01567, 122.02525, 96.02033, 69.00870	uric acid	ZDC
17	1.538	C_5_H_4_O_3_	111.00928 [M − H]^−^	112.01604	68.02179, 67.01842	2-furoic acid	S
18	1.601	C_9_H_12_N_2_O_6_	243.06631 [M − H]^−^	244.06954	200.05885, 153.03059, 152.03633, 140.03606, 110.02486	uridine	ZDC, S
19	1.645	C_6_H_5_NO_3_	138.02071 [M − H]^−^	139.02694	95.03307, 94.02973	6-hydroxypicolinic acid	ZDC
20	1.708	C_6_H_6_O_6_	173.01114 [M − H]^−^	174.01644	129.01999, 111.00896, 101.02464, 85.02927, 83.01366, 59.01327	1,2,3-cyclopropanetricarboxylic acid	S
21	1.747	C_5_H_8_O_5_	147.03131 [M − H]^−^	148.03717	129.01993, 101.02454, 99.00903, 85.02927, 71.01347, 59.01328	D-ribono-1,4-lactone	S
22	1.775	C_7_H_6_O_4_	153.02077 [M − H]^−^	154.02661	110.03291, 109.02972	protocatechuic acid	S
23	1.842	C_4_H_6_O_4_	117.01985 [M − H]^−^	118.02661	73.02905, 71.01340, 59.01314, 55.01809	methylmalonic acid	Z, YY, ZDC, S
24	2.030	C_6_H_12_O_6_	179.05832 [M − H]^−^	180.06339	161.04662, 119.03529, 101.02451, 89.02423, 71.01341, 59.01326	D-(+)-mannose	S
25	2.273	C_5_H_6_O_4_	129.02031 [M − H]^−^	130.02661	85.02927	citraconic acid	S
26	2.541	C_6_H_6_O_3_	125.02518 [M − H]^−^	126.03169	83.01349, 81.03407, 57.03392	phloroglucinol	YY, ZDC
27	2.862	C_7_H_6_O_5_	169.01601 [M − H]^−^	170.02152	125.02486, 97.02945, 81.03418, 69.03392	gallic acid	Z, YY, ZDC, S
28	2.864	C_6_H_6_O_3_	125.02513 [M − H]^−^	126.03169	97.02933, 59.01316	pyrogallol	Z, YY, ZDC, S
29	2.894	C_7_H_6_O_4_	153.02032 [M − H]^−^	154.02661	110.03296, 109.02961, 67.01838	3,5-dihydroxybenzoic acid	Z, YY, ZDC, S
30	4.706	C_7_H_6_O_4_	153.02036 [M − H]^−^	154.02661	152.01241	2,3-dihydroxybenzoic acid	Z, YY, ZDC, S
31	4.738	C_8_H_8_O_4_	167.03642 [M − H]^−^	168.04226	124.04897, 123.04552	6-methoxysalicylic acid	Z, YY
32	5.469	C_10_H_10_O_6_	225.04332 [M − H]^−^	226.04774	135.04541, 121.02985, 109.02958, 59.01313	(1,3-phenylenedioxy) diacetic acid	Z, YY, ZDC
33	5.754	C_9_H_10_O_3_	165.05710 [M − H]^−^	166.06299	150.03308, 123.04549, 122.03756	3′,4′-dihydroxyphenylacetone	YY
34	5.995	C_7_H_6_O_4_	153.02031 [M − H]^−^	154.02661	109.02957, 108.02189, 81.03415	gentisic acid	Z, YY, ZDC, S
35	8.990	C_7_H_6_O_3_	137.02521 [M − H]^−^	138.03169	136.01724, 109.02949	2,5-dihydroxybenzaldehyde	Z, YY, ZDC, S
36	9.135	C_15_H_16_O_9_	339.07727 [M − H]^−^	340.07943	177.02081, 176.01295, 133.03027	esculin	ZDC
37	9.915	C_7_H_6_O_3_	137.02522 [M − H]^−^	138.03169	93.03439, 65.03909	salicylic acid	Z, YY
38	11.505	C_7_H_6_O_3_	137.02502 [M − H]^−^	138.03169	93.03434	3-hydroxybenzoic acid	Z, YY
39	11.511	C_8_H_8_O_4_	167.03632 [M − H]^−^	168.04226	166.02802, 152.01221, 123.04551, 109.02940, 81.03425	vanillic acid	YY
40	11.624	C_15_H_14_O_7_	305.07040 [M − H]^−^	306.07394	219.06837, 167.03592, 137.02496, 125.02476	epigallocatechin	Z, YY, ZDC, S
41	12.790	C_15_H_14_O_6_	289.07547 [M − H]^−^	290.07904	245.08464, 205.05247, 179.03635, 151.04076, 137.02495, 125.02473	catechin	Z, YY, ZDC, S
42	11.840	C_8_H_8_O_3_	151.04120 [M − H]^−^	152.04734	109.02956	resorcinol monoacetate	Z, YY
43	12.505	C_8_H_8_O_4_	167.03650 [M − H]^−^	168.04226	152.01231, 123.04549, 108.02176, 91.01878, 81.03414	methyl protocatechuate	YY
44	13.250	C_9_H_6_O_4_	177.02065 [M − H]^−^	178.02661	133.03004, 105.03464	esculetin	Z, YY, ZDC
45	13.257	C_7_H_6_O_2_	121.02996 [M − H]^−^	122.03678	108.02162, 93.03463, 61.98763	benzoic acid	Z, YY, ZDC, S
46	14.036	C_9_H_8_O_4_	179.03674 [M − H]^−^	180.04226	135.04575, 134.03816, 107.05027, 93.03451	2,5-dihydroxycinnamic acid	Z, YY
47	14.335	C_16_H_18_O_10_	369.08844 [M − H]^−^	370.09000	354.06393, 207.03201, 206.02415, 192.00818, 191.00055	fraxin	ZDC
48	15.978	C_27_H_30_O_14_	577.14227 [M − H]^−^	578.16357	407.08203, 289.07550, 245.08484, 161.02521, 125.02476	kaempferitrin	Z, YY, ZDC
49	17.256	C_15_H_12_O_8_	319.03960 [M − H]^−^	320.05267	193.01591, 175.00494, 151.00441, 125.02476	dihydromyricetin	Z, YY, ZDC, S
50	17.334	C_10_H_8_O_5_	207.03236 [M − H]^−^	208.03717	192.00827, 175.00537, 164.01242, 123.00889, 120.02198, 108.02187	fraxetin	Z, YY
51	17.604	C_15_H_14_O_6_	289.07550 [M − H]^−^	290.07904	245.08466, 205.05249, 179.03644, 151.04080, 137.02499, 125.02477	epicatechin	Z, YY, ZDC, S
52	18.084	C_21_H_22_O_11_	449.11591 [M − H]^−^	450.11621	287.06018, 269.04935, 259.06467, 243.06870, 179.00037, 125.02494	dihydrokaempferol-7-*O*-*β*-d-glucopyranoside	Z, YY, ZDC
53	18.476	C_9_H_10_O_5_	197.04768 [M − H]^−^	198.05282	123.00931	syringic acid	Z, YY
54	18.573	C_8_H_8_O_4_	167.03664 [M − H]^−^	168.04226	153.01599, 152.01239, 123.04564, 108.02164	5-methoxysalicylic acid	Z, ZDC
55	19.379	C_9_H_8_O_4_	179.03677 [M − H]^−^	180.04226	166.02824, 151.00458, 109.02962	caffeic acid	YY
56	20.056	C_15_H_12_O_6_	287.06024 [M − H]^−^	288.06339	259.06451, 243.06905, 201.05765, 125.02485	dihydrokaempferol	Z, YY, ZDC
57	21.943	C_15_H_14_O_5_	273.08102 [M − H]^−^	274.08358	229.08942, 205.08894, 189.05740, 137.02499, 97.02942	epiafzelechin	Z, YY, ZDC
58	23.187	C_21_H_20_O_13_	479.08969 [M − H]^−^	480.09039	317.03189, 316.02646, 271.02771, 179.00029, 151.00427	myricetin-3-*O*-*β*-d-galactopyranoside	Z, YY, ZDC
59	24.958	C_8_H_8_O_4_	167.03621 [M − H]^−^	168.04226	151.00465, 125.02480, 123.04556, 81.03425	2,4,6-trihydroxyacetophenone	Z, YY
60	26.457	C_15_H_12_O_7_	303.05502 [M − H]^−^	304.05829	285.04410, 177.02061, 125.02477	dihydroquercetin	Z, YY, ZDC, S
61	30.264	C_21_H_20_O_12_	463.09525 [M − H]^−^	464.09548	301.03836, 300.03162, 271.02808, 255.03275, 179.00040, 151.00453	quercetin-3-*O*-*β*-d-glucoside	Z, YY
62	31.091	C_9_H_10_O_4_	181.05251 [M − H]^−^	182.05791	153.02026, 152.01241, 109.02968	DL-4-hydroxyphenyllactic acid	Z, YY
63	31.500	C_30_H_24_O_12_	575.12653 [M − H]^−^	576.12622	449.09335, 423.07751, 289.07559, 285.04425, 245.08495, 125.02482	proanthocyanidin A2	Z, YY, ZDC
64	32.618	C_9_H_16_O_4_	187.09972 [M − H]^−^	188.10486	169.08818, 143.10840, 125.09764, 97.06590	azelaic acid	Z, YY
65	33.737	C_15_H_12_O_7_	303.05542 [M − H]^−^	304.05775	151.00447, 125.02480, 107.01392	(2R,3R)-3,3′,5,5′,7-pentahydroxydihydroflavone	Z, YY, ZDC
66	34.608	C_10_H_12_O_5_	211.02725 [M − H]^−^	212.06793	179.00015, 151.00444	3,4,5-trimethoxy benzoic acid	Z, YY, ZDC, S
67	35.002	C_10_H_8_O_4_	191.03723 [M − H]^−^	192.04226	147.04634, 143.86595, 111.00893	7,8-dihydroxy-4-methylcoumarin	YY
68	36.413	C_36_H_54_O_13_	693.34467 [M − H]^−^	694.35644	161.04663, 143.03580, 131.03590, 113.02465, 101.02452	apobioside	ZDC
69	37.539	C_15_H_10_O_8_	317.03439 [M − H]^−^	318.03757	289.03931, 179.00020, 151.00458, 137.02512, 107.01401	myricetin	Z, YY, ZDC, S
70	39.059	C_15_H_10_O_7_	301.04001 [M − H]^−^	302.04265	300.03204, 151.00447, 149.02492	quercetin	Z, YY, ZDC, S
71	39.266	C_14_H_12_O_3_	227.07462 [M − H]^−^	228.07864	185.06258, 183.08304, 157.06697, 143.05128	resveratrol	YY

**Table 2:** Migratory components in the blood of various extracts of Xanthocerais lignum.

No	t_R_/min	Molecular Formula	Actual Measured *M*_r._	Theoretical Exact *M*_r._	Secondary Mass Spectral Fragmentation	Compounds	Source
1	1.023	C_2_H_7_NO_3_S	124.00883 [M − H]^−^	125.01466	79.95736	taurine	YY
2	1.067	C_5_H_4_N_4_O_3_	167.02339 [M − H]^−^	168.02834	125.01845, 124.01601, 122.02512, 97.00449, 96.02065, 69.00910	uric acid *	Z
3	1.072	C_5_H_7_NO_3_	128.03680 [M − H]^−^	129.04259	84.04539, 82.02987	4-oxoproline *	Z, YY, ZDC, S
4	1.118	C_6_H_12_O_7_	195.05443 [M − H]^−^	196.05830	177.04272, 129.02052, 99.00924, 87.00890, 75.00870, 59.01346	gluconic acid	YY
5	1.152	C_6_H_8_O_7_	191.02295 [M − H]^−^	192.02700	129.02043, 111.00939, 87.00887, 85.02956, 59.01345	citric acid *	YY
6	1.509	C_4_H_6_O_5_	133.01605 [M − H]^−^	134.02152	115.00466, 89.02476, 87.00907, 72.99312, 71.01383	DL-malic acid *	Z, YY, ZDC, S
7	1.698	C_6_H_12_O_6_	179.05872 [M − H]^−^	180.06339	119.03571, 113.02481, 101.02478, 89.02443, 71.01356, 59.01340	D-(+)-glucose *	Z
8	1.720	C_4_H_6_O_4_	117.02027 [M − H]^−^	118.02661	73.02927, 71.01345, 59.01336, 55.01847	methylmalonic acid *	Z, YY, S
9	1.778	C_5_H_8_O_5_	147.03152 [M − H]^−^	148.03717	129.02020, 89.02441, 85.02943, 59.01340	D-ribono-1,4-lactone *	Z, YY
10	2.314	C_8_H_9_NO_4_	182.04890 [M − H]^−^	183.05316	138.05753, 108.04621	4-pyridoxic acid	ZDC
11	2.891	C_7_H_6_O_5_	169.01643 [M − H]^−^	170.02152	125.02541, 124.01739, 97.02994, 81.03459, 69.03433	gallic acid *	Z, YY
12	2.960	C_9_H_11_NO_2_	164.07390 [M − H]^−^	165.07898	147.04666, 118.06743, 103.05579, 91.05550, 72.00897	L-phenylalanine	Z, YY, ZDC, S
13	3.408	C_10_H_14_N_2_O_5_	241.08748 [M − H]^−^	242.09027	151.05276, 125.03672	thymidine	YY
14	6.943	C_11_H_12_N_2_O_2_	203.08595 [M − H]^−^	204.08988	186.05890, 159.09451, 142.06769, 116.05137, 74.02470, 72.00899	D-(+)-tryptophan	ZDC, S
15	7.670	C_9_H_10_O_4_	181.05328 [M − H]^−^	182.05791	163.04182, 135.04643, 119.05105	DL-4-hydroxyphenyllactic acid *	Z, YY, ZDC
16	8.061	C_6_H_6_O_4_S	172.99287 [M − H]^−^	173.99868	109.02998, 93.03465, 79.95716	4-phenolsulfonic acid	Z, YY, ZDC, S
17	11.550	C_15_H_14_O_7_	305.07166 [M − H]^−^	306.07394	219.06976, 179.03711, 167.03688, 137.02544, 125.02546	epigallocatechin *	YY
18	11.547	C_21_H_22_O_12_	465.11096 [M − H]^−^	466.11058	289.07651, 245.08553, 137.02544, 113.02486, 85.02943	epicatechin-3′-*O*-glucuronide	Z, YY
19	12.123	C_8_H_7_NO_4_S	212.00600 [M − H]^−^	213.00958	132.04686, 120.04624, 118.03075, 80.96530, 79.95746	3-indoxyl sulphate	YY, ZDC, S
20	12.710	C_15_H_14_O_6_	289.07669 [M − H]^−^	290.07904	245.08562, 203.07404, 179.03705, 125.02530, 109.02999	catechin *	Z, YY
21	14.693	C_8_H_15_NO_3_	172.10043 [M − H]^−^	173.10519	130.08870, 128.10936, 82.06647, 58.02952	2-(acetylamino)hexanoic acid	Z, YY, ZDC
22	17.240	C_15_H_12_O_8_	319.05203 [M − H]^−^	320.05267	193.01700, 175.00581, 151.00520, 125.02543	dihydromyricetin *	YY
23	17.766	C_15_H_14_O_6_	289.07736 [M − H]^−^	290.07904	245.08604, 205.05362, 179.03735, 137.02571, 125.02541, 109.03013	epicatechin *	Z, YY
24	18.959	C_22_H_24_O_12_	479.12704 [M − H]^−^	480.12623	303.09225, 175.02661, 137.02541, 113.02486, 85.02940	4′-*O*-methyl-epicatechin-3′-*O*-glucuronide	Z, YY, ZDC
25	19.246	C_11_H_13_NO_3_	206.08531 [M − H]^−^	207.08954	164.07327, 147.04642, 91.05540, 70.02953, 58.02937	(R,Z)-2-[(1-hydroxyethylidene)amino]-3-phenylpropanoic acid	Z
26	19.685	C_9_H_8_O_3_	163.04227 [M − H]^−^	164.04734	119.05114, 93.03474, 91.05582	2,3-dihydro-1-benzofuran-2-carboxylic acid	YY, ZDC
27	23.110	C_11_H_11_NO_3_	204.06943 [M − H]^−^	205.07389	186.05820, 158.06247, 142.06714, 116.05107, 72.99293	indole-3-lactic acid	Z
28	24.607	C_9_H_7_NO	144.04698 [M − H]^−^	145.05276	116.05098	4-indolecarbaldehyde	Z
29	26.537	C_15_H_12_O_7_	303.05502 [M − H]^−^	304.05829	285.04410, 177.02061, 125.02477	dihydroquercetin *	Z, YY
30	30.011	C_21_H_18_O_13_	477.07565 [M − H]^−^	478.07474	301.04028, 179.00060, 151.00490, 121.03023, 113.02496, 71.01352	quercetin-3-*O*-glucuronide	Z
31	31.777	C_30_H_24_O_12_	575.12909 [M − H]^−^	576.12622	285.04523, 245.08562, 125.02518	proanthocyanidin A2 *	Z, YY
32	32.619	C_9_H_16_O_4_	187.10034 [M − H]^−^	188.10486	169.08893, 143.10909, 125.09803, 97.06611, 57.03410	azelaic acid *	Z, YY, ZDC
33	33.302	C_10_H_10_O_4_	193.05389 [M − H]^−^	194.05791	178.03018, 137.02583, 134.03888	isoferulic acid	ZDC
34	34.017	C_21_H_22_O_9_	417.12589 [M − H]^−^	418.12583	135.04620, 119.05073, 91.76878	liquiritin	Z, YY, ZDC, S
35	35.872	C_22_H_20_O_13_	491.09137 [M − H]^−^	492.08984	315.05624, 300.03238, 113.02492	isorhamnetin-3-*O*-glucuronide	Z, YY
36	37.760	C_15_H_10_O_8_	317.03574 [M − H]^−^	318.04161	179.00099, 151.00528, 137.02577, 107.01434	myricetin *	YY

Note: * denotes prototype blood-entering components.

**Table 3:** Results of gray correlation analysis.

Constituents Absorbed into Blood	Correlation	Rank
azelaic acid	0.924	1
DL-4-hydroxyphenyllactic acid	0.871	2
proanthocyanidin A2	0.846	3
D-ribono-1,4-lactone	0.844	4
isorhamnetin-3-*O*-glucuronide	0.842	5
epicatechin-3′-*O*-glucuronide	0.839	6
epicatechin	0.837	7
4′-*O*-methyl-epicatechin-3′-*O*-glucuronide	0.834	8
dihydroquercetin	0.834	9
catechin	0.832	10
L-phenylalanine	0.763	11
2-(acetylamino)hexanoic acid	0.749	12
liquiritin	0.748	13
4-oxoproline	0.745	14
methylmalonic acid	0.732	15
DL-malic acid	0.717	16
3-indoxyl sulphate	0.706	17
4-phenolsulfonic acid	0.658	18
myricetin	0.653	19
dihydromyricetin	0.653	20
gallic acid	0.653	21
epigallocatechin	0.653	22
gluconic acid	0.653	23
citric acid	0.653	24
thymidine	0.653	25
taurine	0.653	26
2,3-dihydro-1-benzofuran-2-carboxylic acid	0.649	27
uric acid	0.648	28
D-(+)-glucose	0.648	29
quercetin-3-*O*-glucuronide	0.648	30
4-indolecarbaldehyde	0.648	31
indole-3-lactic acid	0.648	32
(R,Z)-2-[(1-hydroxyethylidene)amino]-3-phenylpropanoic acid	0.648	33
4-pyridoxic acid	0.582	34
isoferulic acid	0.582	35
D-(+)-tryptophan	0.582	36

**Table 4:** Molecular docking results.

Compound	Receptor	PDB ID	CDOCKER Interaction Energy (kcal·mol^−1^)
proanthocyanidin A2	ERK	6SLG	−71.2989
	NF-κB	1SVC	(-)
	HIF-1α	1LM8	(-)
	VEGF	5T89	−41.3564
epicatechin	ERK	6SLG	−43.0673
	NF-κB	1SVC	−33.9534
	HIF-1α	1LM8	−7.359
	VEGF	5T89	−26.992
dihydroquercetin	ERK	6SLG	−43.7723
	NF-κB	1SVC	−32.3677
	HIF-1α	1LM8	−9.4884
	VEGF	5T89	−23.7314
catechin	ERK	6SLG	−43.9609
	NF-κB	1SVC	−27.5155
	HIF-1α	1LM8	−14.743
	VEGF	5T89	−29.1681

## Data Availability

The original contributions presented in the study are included in the article/Supplementary Materials; further inquiries can be directed to the corresponding author.

## Supplementary Materials

The following supporting information can be downloaded at https://www.mdpi.com/article/10.3390/molecules29133191/s1, Table S1: Peak areas of migrating components in the blood of various extracts of Xanthocerais lignum; Table S2: Experimentally active targets of Q-Marker; Table S3: Anti-RA efficacy of various extracts of Xanthocerais lignum.

## References

[1] Smolen J.S., Aletaha D., McInnes I.B. (2016). Rheumatoid arthritis. Lancet.

[2] Finckh A., Gilbert B., Hodkinson B., Bae S.C., Thomas R., Deane K.D., Alpizar-Rodriguez D., Lauper K. (2022). Global epidemiology of rheumatoid arthritis. Nat. Rev. Rheumatol..

[3] Rzhepakovsky I., Anusha Siddiqui S., Avanesyan S., Benlidayi M., Dhingra K., Dolgalev A., Enukashvily N., Fritsch T., Heinz V., Kochergin S. (2021). Anti-arthritic effect of chicken embryo tissue hydrolyzate against adjuvant arthritis in rats (X-ray microtomographic and histopathological analysis). Food Sci. Nutr..

[4] Porcello A., Gonzalez-Fernandez P., Jeannerat A., Peneveyre C., Abdel-Sayed P., Scaletta C., Raffoul W., Hirt-Burri N., Applegate L.A., Allémann E. (2023). Thermo-Responsive Hyaluronan-Based Hydrogels Combined with Allogeneic Cytotherapeutics for the Treatment of Osteoarthritis. Pharmaceutics.

[5] Editorial Committee of the Chinese Materia Medica (2004). Chinese Materia Medica (Monk’s Medicine Volume).

[6] Editorial Committee of Flora of China, Chinese Academy of Sciences (2020). Chinese Medicinal Plants (Volume VI).

[7] Zhao Y., Zhao L., Cao R. (2020). Flora of Inner Mongolia (Volume III).

[8] Liu C., Chen S., Xiao X., Zhang T., Hou W., Liao M. (2016). A new concept on quality marker of Chinese materia medica: Quality control for Chinese medicinal products. Chin. Tradit. Herb. Drugs.

[9] Zhu C., Jiang Z., Li J., Zhang B. (2020). Overview research status in serum spectrum-effect of Chinese materia medica. Chin. Tradit. Herb. Drugs.

[10] Wang M., Xia Y., Yang M., Wu D., Liu L., Ge G., Yang L., Hou J. (2015). Advances in study on biosynthsis of O-glucuronides. Chin. Tradit. Herb. Drugs.

[11] Borges G., Ottaviani J.I., van der Hooft J.J.J., Schroeter H., Crozier A. (2018). Absorption, metabolism, distribution and excretion of (-)-epicatechin: A review of recent findings. Mol. Asp. Med..

[12] Azzeh M., Neagu D., Cowling P.I. (2010). Fuzzy grey relational analysis for software effort estimation. Empir. Softw. Eng..

[13] Doi K., Murata K., Ito S., Suzuki A., Terao C., Ishie S., Umemoto A., Murotani Y., Nishitani K., Yoshitomi H. (2022). Role of Lysine-Specific Demethylase 1 in Metabolically Integrating Osteoclast Differentiation and Inflammatory Bone Resorption Through Hypoxia-Inducible Factor 1α and E2F1. Arthritis Rheumatol..

[14] Sadeghi Shaker M., Rokni M., Mahmoudi M., Farhadi E. (2023). Ras family signaling pathway in immunopathogenesis of inflammatory rheumatic diseases. Front. Immunol..

[15] Liu F., Feng X.X., Zhu S.L., Huang H.Y., Chen Y.D., Pan Y.F., June R.R., Zheng S.G., Huang J.L. (2018). Sonic Hedgehog Signaling Pathway Mediates Proliferation and Migration of Fibroblast-Like Synoviocytes in Rheumatoid Arthritis via MAPK/ERK Signaling Pathway. Front. Immunol..

[16] Pinzi L., Rastelli G. (2019). Molecular Docking: Shifting Paradigms in Drug Discovery. Int. J. Mol. Sci..

[17] Trentham D.E., Townes A.S., Kang A.H. (1977). Autoimmunity to type II collagen an experimental model of arthritis. J. Exp. Med..

[18] Liu X., Wang Z., Qian H., Tao W., Zhang Y., Hu C., Mao W., Guo Q. (2022). Natural medicines of targeted rheumatoid arthritis and its action mechanism. Front. Immunol..

[19] Fabre N., Rustan I., de Hoffmann E., Quetin-Leclercq J. (2001). Determination of flavone, flavonol, and flavanone aglycones by negative ion liquid chromatography electrospray ion trap mass spectrometry. J. Am. Soc. Mass Spectrom..

[20] Shi P., Lin X., Yao H. (2018). A comprehensive review of recent studies on pharmacokinetics of traditional Chinese medicines (2014-2017) and perspectives. Drug Metab. Rev..

[21] Li Y., Wang Y., Tai W., Yang L., Chen Y., Chen C., Liu C. (2015). Challenges and Solutions of Pharmacokinetics for Efficacy and Safety of Traditional Chinese Medicine. Curr. Drug Metab..

[22] Zhang F., Gong X., Xiao B., Zhang C., Wang Z. (2013). Pharmacokinetics and tissue distribution of a bioactive sesquiterpenoid from Polygonum jucundum following oral and intravenous administrations to rats. J. Pharm. Biomed. Anal..

[23] Lv G., Lou Z., Chen S., Gu H., Shan L. (2011). Pharmacokinetics and tissue distribution of 2,3,5,4′-tetrahydroxystilbene-2-O-β-D-glucoside from traditional Chinese medicine Polygonum multiflorum following oral administration to rats. J. Ethnopharmacol..

[24] Wang Q., Wen X., Kong J. (2020). Recent Progress on Uric Acid Detection: A Review. Crit. Rev. Anal. Chem..

[25] Akram M. (2014). Citric acid cycle and role of its intermediates in metabolism. Cell Biochem. Biophys..

[26] Froese D.S., Fowler B., Baumgartner M.R. (2019). Vitamin B(12), folate, and the methionine remethylation cycle-biochemistry, pathways, and regulation. J. Inherit. Metab. Dis..

[27] Hazafa A., Rehman K.U., Jahan N., Jabeen Z. (2020). The Role of Polyphenol (Flavonoids) Compounds in the Treatment of Cancer Cells. Nutr. Cancer.

[28] Chen B., Zhou Y., Wang Z., Yang Y., Qiu F., Yan X. (2022). Research progress on biosynthesis of glucuronides of plant natural products. Chin. Tradit. Herb. Drugs.

[29] Natsume M., Osakabe N., Yasuda A., Baba S., Tokunaga T., Kondo K., Osawa T., Terao J. (2004). In Vitro Antioxidative Activity of (−)-Epicatechin Glucuronide Metabolites Present in Human and Rat Plasma. Free Radic. Res..

[30] Hughes S.D., Ketheesan N., Haleagrahara N. (2017). The therapeutic potential of plant flavonoids on rheumatoid arthritis. Crit. Rev. Food Sci. Nutr..

[31] Wang Q.Q., Gao H., Yuan R., Han S., Li X.X., Tang M., Dong B., Li J.X., Zhao L.C., Feng J. (2020). Procyanidin A2, a polyphenolic compound, exerts anti-inflammatory and anti-oxidative activity in lipopolysaccharide-stimulated RAW264.7 cells. PLoS ONE.

[32] Tang L.Q., Wei W., Wang X.Y. (2007). Effects and mechanisms of catechin for adjuvant arthritis in rats. Adv. Ther..

[33] Wu C., Li F., Zhang X., Xu W., Wang Y., Yao Y., Han Z., Xia D. (2022). (−)-Epicatechin Ameliorates Monosodium Urate-Induced Acute Gouty Arthritis Through Inhibiting NLRP3 Inflammasome and the NF-κB Signaling Pathway. Front. Pharmacol..

[34] Zhang H.Q., Wang Y.J., Yang G.T., Gao Q.L., Tang M.X. (2019). Taxifolin Inhibits Receptor Activator of NF-κB Ligand-Induced Osteoclastogenesis of Human Bone Marrow-Derived Macrophages in vitro and Prevents Lipopolysaccharide-Induced Bone Loss in vivo. Pharmacology.

[35] Guo X., Chen G. (2020). Hypoxia-Inducible Factor Is Critical for Pathogenesis and Regulation of Immune Cell Functions in Rheumatoid Arthritis. Front. Immunol..

[36] Li H., Wu Q.Y., Teng X.H., Li Z.P., Zhu M.T., Gu C.J., Chen B.J., Xie Q.Q., Lu O.X. (2023). The pathogenesis and regulatory role of HIF-1 in rheumatoid arthritis. Cent.-Eur. J. Immunol..

[37] Szekanecz Z., Besenyei T., Paragh G., Koch A.E. (2009). Angiogenesis in rheumatoid arthritis. Autoimmunity.

[38] Rashid M., Zadeh L.R., Baradaran B., Molavi O., Ghesmati Z., Sabzichi M., Ramezani F. (2021). Up-down regulation of HIF-1α in cancer progression. Gene.

[39] Yang W., Wei X., Jiao Y., Bai Y., Sam W.N., Yan Q., Sun X., Li G., Ma J., Wei W. (2022). STAT3/HIF-1α/fascin-1 axis promotes RA FLSs migration and invasion ability under hypoxia. Mol. Immunol..

